# Multicancer early detection tests: where are we?

**DOI:** 10.1093/jncics/pkac084

**Published:** 2022-12-01

**Authors:** Holli A Loomans-Kropp

**Affiliations:** Divison of Cancer Prevention and Control, Department of Internal Medicine, College of Medicine, The Ohio State University, Columbus, OH, USA

The holy grail of a highly sensitive and specific multicancer early detection test (MCED) has been a focus of much research over the last several decades. In this issue of *JNCI Cancer Spectrum*, Matsuzaki and colleagues (1) propose an MCED that combines microRNA (miRNA) expression with machine learning to predict the tissue of origin for 13 different cancer types. Using large training and validation sets of cancer and noncancer benign tissues, Matsuzaki et al. demonstrated 88% accuracy in identifying the originating tissue type, with an accuracy of 90% for stage I-II cancer. Several additional studies have investigated the utilization of miRNAs for early cancer detection. A recent meta-analysis found that panels of 3 or more miRNAs could predict colorectal cancer with moderate to good accuracy (area under the curve = 0.70-0.94) (2). A separate study found that a 5 miRNA panel, validated in 3 independent cohorts, showed 81.3% sensitivity for non-small cell lung cancer detection, and an additional study, also using a 5 miRNA risk score, predicted overall lung cancer survival following immunotherapy (3,4). Expression of miR-5100, miR-1343-3p, miR-1290, and miR-4787-3p achieved 99% sensitivity and specificity for lung cancer from prediagnostic samples (5).

The results of the current study feed additional data into the growing field of MCED efficacy, with the collective goal of using molecular signatures to identify and detect asymptomatic cancer using minimally invasive, blood-based techniques for easy, rapid detection, rather than undergoing a series of invasive screening tests at different intervals. The current cancer screening recommendations in the United States state the following:

- Breast cancer screening by biennial mammography for women aged 50 to 74 years (6).
- Cervical cancer screening by cervical cytology and human papilloma virus testing every 3 and 5 years, respectively, dependent on age (7).
- Colorectal cancer screening by fecal occult blood test or fecal immunochemical test (FOBT or FIT) annually, stool DNA-FIT every 1-3 years, computed tomography (CT) colonography every 5 years, flexible sigmoidoscopy every 5 years (if adding FIT, the interval extends to every 10 years), or colonoscopy every 10 years for individuals aged 45 to 74 years (8,9). This recommendation statement was recently revised by the US Preventive Services Task Force to reflect the increasing incidence of early onset colorectal cancer.
- Lung cancer screening by low-dose CT for individuals with increased risk of lung cancer development (aged 50 to 80 years with current and former smokers with 20 pack-year smoking history) (10).

Simplifying the screening process to a single initial test, or decreasing the number of individual screening tests, could have a notable public health impact, perhaps improving compliance because of reduced burden placed on the individual—fewer appointments, no preparatory procedures. Considering the progress made in MCED development, many questions remain that must be answered before MCEDs can be widely implemented. Most of the currently published studies establishing MCED sensitivity and specificity evaluated samples from individuals with known, preexisting cancer. For example, the Cell-free Genome Atlas study was a multicenter study that evaluated the efficacy of methylation-based cell-free DNA markers, in combination with artificial intelligence, to discern between known cancer cases and noncancer controls with moderate sensitivity (51.5%) (11). An additional test, PanSeer, uses a 277 circulating tumor DNA methylation marker panel for the detection of colorectal, esophageal, liver, lung, or stomach cancer (12). Though initially tested in a case-control setting, the investigators prospectively collected blood from asymptomatic individuals and found a 95% cancer detection rate up to 4 years prior to diagnosis, though the authors acknowledge the possibility of assay misclassification, most likely from the presence of an undetected, undiagnosed primary cancer.

Another meaningful consideration for the implementation of MCEDs on a large scale is the issue of follow-up. In the event an individual receives a positive MCED result, what is the protocol for physician or individual follow-up? Timely follow-up has already proven an issue with conventional screening methods, such as FOBT/FIT. The Population-Based Research Optimizing Screening through Personalized Regimens consortium found that only 68% of individuals with an abnormal FOBIT/FIT received follow-up care within 3 months of a positive test (13). A recent systematic analysis of FOBT or FIT included in observational studies or randomized clinical trials showed that colorectal cancer incidence increased sharply if individuals didn’t undergo colonoscopy until 12 months or later following a positive FOBT or FIT test (14). MCEDs pose a greater challenge, as follow-up is dependent on positive tissue of origin (if determined), rather than a streamlined process designated for an individual cancer type, such as FOBT/FIT to colonoscopy. Furthermore, what is the appropriate response for when an individual goes for a follow-up screen after a positive MCED but no cancer is found? The Detecting cancers Earlier Through Elective mutation-based blood Collection and Testing study, a prospective study of approximately 10 000 individuals using CancerSEEK for cancer detection, conducted rigorous follow-up of participants with positron emission tomography (PET)–CT or other imaging techniques for test confirmation; PET-CT confirmation was conducted for 95% of those with a positive test (15). Of those who underwent imaging, only 50% had a concerning result, and 41% had a biopsy-confirmed or other confirmed cancer. On a population scale, however, completing a PET-CT or other imaging test for all MCED-positive individuals is not feasible, particularly in low-resource areas. Not only are standardized procedures needed for immediate follow-up after a positive test but also (1) continued assessment intervals after a positive MCED but no cancer is detected and (2) interval testing after a negative test. To address these questions, a pragmatic, prospective trial of 140 000 asymptomatic individuals is currently enrolling throughout England, with the goal of evaluating the efficacy of the blood-based MCED Galleri (produced by GRAIL) to detect cancer in the general population (16). This study will provide some much needed answers to the posed questions, but completion of this study and the subsequent data analysis is far off. Moreover, health-care policy and larger-scale infrastructure will need improvement to support the implementation of MCEDs into regular practice. Nadauld and Goldman (17) addressed these issues in a recent commentary, stating the continued importance of “regulators, payers, and experts” to play pivotal roles in advancing these new approaches to cancer detection.

MCEDs represent an important advancement in the field of cancer early detection. With our increasing understanding of the molecular mechanisms involved in cancer initiation and progression, and how these mechanisms differ between cancers, developing highly sensitive and specific multiplexed, multicancer blood-based detection tests is within the realm of possibility. This concept, using a panel in miRNAs for determination of cancer tissue-of-origin, was nicely demonstrated by Matsuzaki and colleagues in this issue of *JNCI Cancer Spectrum* (1). However, many questions still need to be addressed before these tests can be employed reliably and on a large scale. With the heightened awareness of the current limitations to MCEDs, many institutions, professional organizations, and investigators are working to tackle these issues as quickly and thoroughly as possible. The age of the MCED for cancer early detection is upon us. Are we ready?

## Data Availability

No new data were generated or analysed in support of this research.

## Notes

**Role of the funder:** Not applicable.

**Author disclosures:** HLK has no disclosures.

**Author contributions:** HLK: writing—original draft, writing—review and editing.

**Disclaimers:** The thoughts and opinions expressed in this editorial are the author’s own and do not reflect those of The Ohio State University.
